# Optofluidics Refractometers

**DOI:** 10.3390/mi9030136

**Published:** 2018-03-20

**Authors:** Cheng Li, Gang Bai, Yunxiao Zhang, Min Zhang, Aoqun Jian

**Affiliations:** 1 State Key Laboratory of Information Photonics and Optical Communications, Beijing University of Posts and Telecommunications, No. 10, Xitucheng Road, Haidian District, Beijing 100876, China; lichn@bupt.edu.cn (C.L.); mzhang@bupt.edu.cn (M.Z.); 2 MicroNano System Research Center, College of Information and Computer Science, Taiyuan University of Technology, Taiyuan 030024, China; baigang0308@link.tyut.edu.cn (G.B.); zhangyunxiao1895@link.tyut.edu.cn (Y.Z.); 3 Key Laboratory of Advanced Transducers and Intelligent Control System, Shanxi Province and Ministry of Education, Taiyuan 030024, China

**Keywords:** refractometry, refractive index, microfluidics

## Abstract

Refractometry is a classic analytical method in analytical chemistry and biosensing. By integrating advanced micro- and nano-optical systems with well-developed microfluidics technology, optofluidics are shown to be a powerful, smart and universal platform for refractive index sensing applications. This paper reviews recent work on optofluidic refractometers based on different sensing mechanisms and structures (e.g., photonic crystal/photonic crystal fibers, waveguides, whisper gallery modes and surface plasmon resonance), and traces the performance enhancement due to the synergistic integration of optics and microfluidics. A brief discussion of future trends in optofluidic refractometers, namely volume sensing and resolution enhancement, are also offered.

## 1. Introduction

Refractive index (RI), a basic physical substance property, can be used to measure solute concentration and purity in transparent liquor such as Salinity and Brix. RI is highly sensitive and precise, enabling it to monitor extreme variations in tiny particles in solution, which can be used to quantitatively analyze chemical components. For example, 10^−9^ RIU is equivalent to 1 femto mol/L of salt in water. Compared to other measuring methods, RI measurement does not actually affect the properties of the analyst and offers real-time, convenient analysis of liquid composition (e.g., label-free analysis of various bio-samples, including DNA and protein). Therefore, refractometry with ultra-high sensitivity has great potential in environmental protection [1,2,3], drinking water safety [4,5] and biomedical applications [6].

Microfluidics has achieved great progress recently due to its own excellent performance in fluidic handling [7,8,9], micro-environment control [10,11,12] and signal amplification [13,14]. By integrating advanced micro- and nano-optical systems with well-developed microfluidics technology, optofluidics has ushered in a new era of lab-on-a-chip functionality [15,16,17,18,19,20,21,22], including biochemical sensing with optical measurement [23], optofluidic imaging [24], and light-driven manipulation [25,26]. In the case of RI sensing, many valuable review papers such as Fan’s [27] have found that synergistic integration creates unique characteristics that promote the performance and function of biological/chemical analysis. First, in some well-designed structures, the analyte can be selectively delivered to a location with maximum light-analyte interaction, which can significantly enhance sensors’ sensitivity and resolution. Second, an extremely small analyte volume (i.e., nL) and some related treatments of biological samples, such as cultivating, sorting, trapping, and purification, can be achieved with microfluidics technology. Other analytical methods, including chromatography, electrophoresis, and Raman scattering, can be cascaded with the RI sensing function to carry out complex analysis. Third, issues related to integration, namely alignment and packaging, can be easily solved, and the device’s volume can be reduced substantially. Moerover, device commercialization will facilitate the development of portable, cost-effective, and highly sensitive bio/chemical analysis instruments. 

In this paper, recent work pertaining to optofluidic refractometers, based on different sensing mechanisms and structures, is sequentially reviewed. Particularly, this review focuses on the brilliant designs that the optical sensing structures, based on their own various characteristics, synergistically integrated with microfluidics to optimize their sensing performances. These designs enhance the resolutions of the sensors, expand the analysis functions of the sensors or solve issues of sensor applications. The revolution tracks of this integration, showing the elegance of the device design, are also presented. A discussion regarding the field’s ongoing development is also offered with the hope to inspire more new ideas from the readers. 

## 2. RI Sensing and Technologies 

### 2.1. Photonic Crystal Fibers

**Figure 1:**
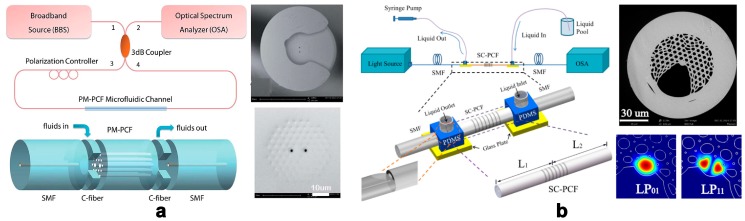
(**a**) Scheme and transverse section graph of the SMF-C-PCF-C-SMF microfluidic device. Reprinted with permission from [30]. Copyright (2014) RSC; (**b**) Scheme of the in-line optofluidic sensing platform and SEM image of the SC-PCF and Simulated intensity distribution of LP_01_ mode and LP_11_ mode at the wavelength of 1550 nm. Reprinted with permission from [31]. Copyright (2016) OSA.

As a classic photonic structure, photonic crystal fibers (PCFs) appear to be an ideal platform for the realization of novel optofluidic sensors [28]. P. Domachuk et al. proposed a compact refractometer utilizing a Fabry–Pérot cavity (FPC) etalon formed between two aligned Bragg grating fibers (one-dimensional PCFs), which located on either side of the microfluidic channel to contain the fluid to be tested [15]. The resolution of the RI sensor based on PCFs can be further improved if the microstructured architecture in the PCFs is filled by analyte [29]. Its waveguide nature ensures strong light-analyte interaction along all PCFs. Some smart structures are designed to feed the analyte into the PCFs’ hollow gap. Many research efforts contribute to design the deliberate structures to achieve the convenient load of liquid sample, and employ special modes to improve the sensitivity of the sensors. For example, C. Wu et al. presented the fabrication and characterization of an in-line photonic crystal fiber microfluidic refractometer outfitted with a C-shaped fiber [30] (Figure 1a). The C-shaped fiber, placed between the PCF and single-mode fiber, achieved two functions simultaneously: in-line optical signal coupling and analyte fluid feeding. Using an arc discharge pre-treatment technique, small air hole voids near the surface were sealed, so only the two central large air hole channels were employed for RI sensing; thus, device sensitivity increased by 70% due to higher power density. Similarly, N. Zhang et al. utilized a side-channel photonic crystal fiber with side-polished single mode fibers to form optofluidic microchannels [31] (Figure 1b). A long-period grating combined with intermodal interference between LP_01_ and LP_11_ core modes was used to sense the liquid’s RI in the side channel.

### 2.2. Planar Optical Waveguides

**Figure 2:**
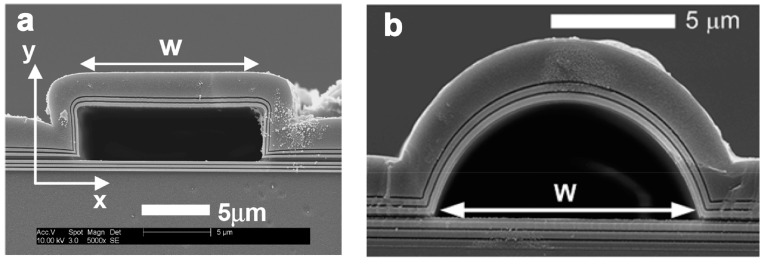
Scanning electron microscope (SEM) images of hollow-core anti-resonant reflecting optical waveguides (ARROWs) with (**a**) rectangular and (**b**) arch-shaped cross sections fabricated by surface micromachining process. Reprinted with permission from [33]. Copyright (2005) OSA.

**Figure 3:**
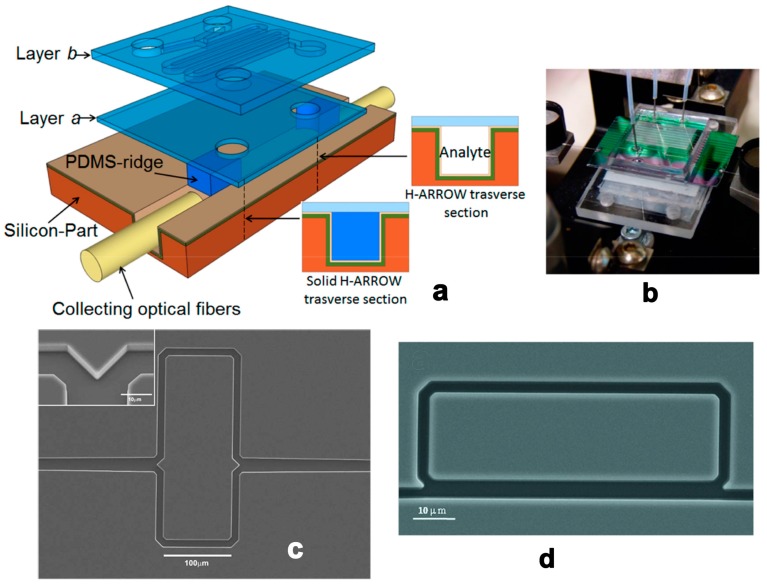
Schematic (**a**) and fabricated chip (**b**) of the sensor based on hybrid ARROW optofluidic platform. Reprinted with permission from [34]. Copyright (2014) OSA; (**c**) SEM picture of liquid-core ARROW. Reprinted with permission from [36]. Copyright (2010) OSA; (**d**) SEM picture of the integrated silicon optofluidic ring resonator. Reprinted with permission from [37]. Copyright (2010) AIP.

An integrated planar optical waveguide (POW) has been utilized for RI sensing for several decades [32]. In this type of sensor, light propagates along the solid waveguide, around which an evanescent field interacts with the analyte to induce phase shift or intensity variation. The light-analyte interaction is naturally limited by the evanescent field’s range per unit length. Therefore, confining light in the fluidic waveguide is an effective way to achieve strong light-analyte interaction. The liquid core anti-resonant reflecting optical waveguide (ARROW) is a novel photonic structure with tightly integrated optical and fluidic structures [33] (Figure 2). Particularly, Campopiano et al. firstly demonstrated a bulk refractometer based on multimode liquid ARROW structure [34] (Figure 3a,b). And G. Testa et al. offered a comprehensive review of an ARROW-based device’s operation principles and applications [35]. Furthermore, the RI microsensor’s sensitivity can be improved by using the ARROW waveguide as interferometer arms [36] (Figure 3c) and part of the ring resonators [37] (Figure 3d). In addition, the waveguide can act as a carrier, on which 2D materials can be deposited, for novel RI sensors developments. Surface plasmon waves or evanescent wave are tuned by the liquid medium on the surface of Graphene [38] and MoS_2_ [39] to realize RI sensing. 

### 2.3. Whisper Gallery Mode

**Figure 4:**
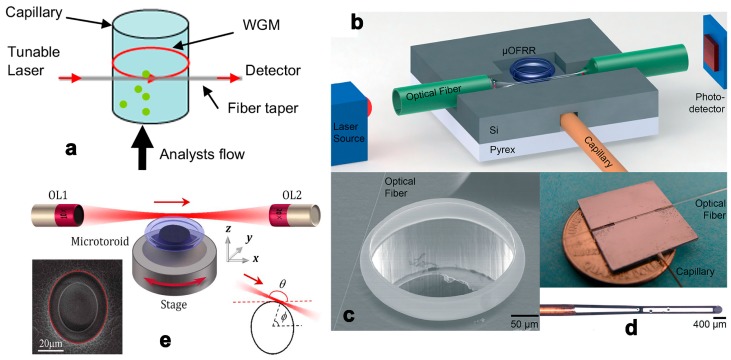
(**a**) The optofluidic ring resonator based on thin-walled capillary. Reprinted with permission from [42]. Copyright (2014) Elsevier; Schematic diagram (**b**), SEM image (**c**) and photograph (**d**) of the μOFRR sensor. Reprinted with permission from [43]. Copyright (2014) RSC; (**e**) Schematic diagram of the experimental setup for free-space coupling between a laser beam and a deformed toroidal microcavity. Reprinted with permission from [44]. Copyright (2016) OSA.

Since F. Vollmer piloted the application of whisper gallery mode (WGM) in protein detection, continued efforts have explored the detection resolution’s potential [40]. A series of well-designed microtoroid resonators were fabricated by L. Yang and Y.-F. Xiao, and related intelligent sensing schemes and noise control methods have also been proposed and optimized [41]. Due to the resonators’ ultra-high Q values, the microsensors have an extremely low detection limit; single nanoparticles have been detected successfully. However, when applying a typical cavity-taper coupling system in an optofluidic system, uncontrollable analyte flow in the microchip may exert a negative effect on cavity-taper coupling, diminishing the Q value to some extent. Thin-wall cylindrical capillaries with spatial analyte-taper separation comprise an alternative scheme that is more convenient for optofluidic integration [42,43] (Figure 4a–d). Since high-Q WGMs in deformed microcavities can be excited by free space coupling [44] (Figure 4e), it is a feasible way to achieve high cavity coupling efficiency in microfluidic chips. 

### 2.4. Surface Plasmon Resonance and Localized Surface Plasmon Resonance

**Figure 5:**
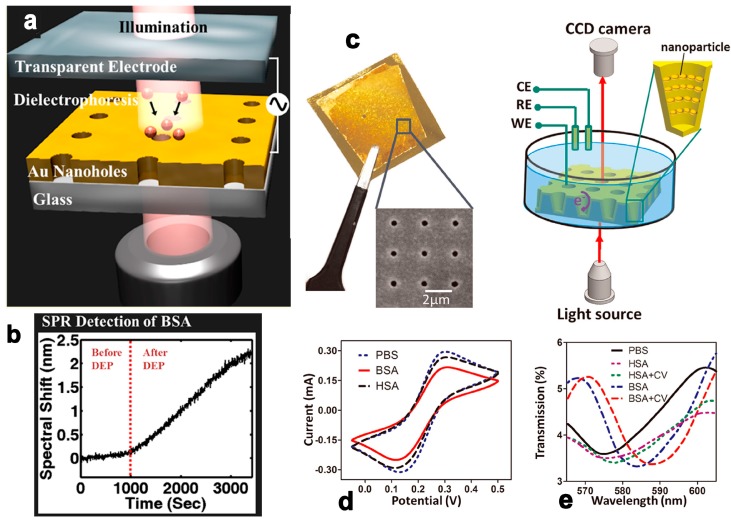
Schematic of the experimental setup for dielectrophoretic concentration of analyte molecules (**a**) and spectral shift of BSA’s SPR detection (**b**). Reprinted with permission from [47]. Copyright (2014) ACS; The nanoCA for electrochemical and LSPR measurement (**c**) and Transmission spectrum (**d**) of nanoCA with PBS, 100 μg/mL HSA and HSA plus CV scanning, 100 μg/mL BSA and BSA plus synchronous CV scanning and statistic (**e**) for shifts in dip wavelength of HSA, HSA plus CV, BSA and BSA plus CV. Reprinted with permission from [49]. Copyright (2015) Elsevier.

Surface plasmon resonance/localized surface plasmon resonance (SPR/LSPR) refer to the excitation of collective electron charge oscillations on planar metal surfaces or onto the surface of metallic nanoparticles by incident light. The oscillation leads to a wavelength-dependent reduction in the overall reflection, which presents an absorption peak in the reflection/scatter spectrum. As the SPR is very sensitive to the RI near the metal surface (within 300 nm), it is widely used to develop RI sensors [45] and biosensors [46]. Unlike most other types of sensing schemes, metal plane/particle/nanohole arrays represent an indispensable part of the sensing system, which also offers unique opportunities for function appending and upgrading. A. Barik et al. utilized a gold nanohole array-based optofluidic device for label-free detection of analyte molecules, of which the nanohole array also generated gradient dielectrophoretic force to accumulate the measured biological analytes [47] (Figure 5a,b). Real-time detection was over 1000 times faster than the classic diffusion method for 1 pM analyte concentration. S. Kang et al. combined RI sensing with surface-enhanced Raman spectroscopy (SERS) in silver–gold layered bimetallic plasmonic crystals to conduct quantitative and qualitative measurements simultaneously [48]. D. Zhang et al. developed a unique nanoscale cup array (nanoCA) coupling electrochemistry to LSPR spectroscopy measurement, offering a novel method by which to evaluate complex electrochemical reaction processes [49] (Figure 5c–e).

## 3. Discussion and Outlook

### 3.1. Parameters for Sensor Characterization

Sensitivity, defined as the magnitude in shift of the characteristic wavelength versus the change in a sample’s RI, is a key parameter describing RI sensor performance. However, for measurement response down to the sensor’s detection limit (i.e., resolution), many factors should be considered: the shape of the resonant peak, noise sources, and signal intensity, among others. According to X. D. Fan’s detailed analysis, a sensor with lower sensitivity but a sharper resonance peak (i.e., a higher-quality factor) has higher resolution [50]. Thus, to accurately describe sensor performance, a comprehensive evaluation that includes sensitivity and quality factors is crucial. Several novel parameters, such as figure of merit (FOM) [51] or detectivity [52], have been proposed in some papers. FOM is defined as the ratio of sensitivity to full wave at half maximum (FWHM) as Equation (1), which can take both of the sensitivity and Q value into consideration, thus the performance of the sensor can be characterized accurately by using a single parameter.

(1) $$\mathrm{FOM}=\frac{\mathrm{Sensitivity}}{\mathrm{FWHM}}$$

Table 1 summarized the critical parameters for qualifying the performances of the RI sensors based on different principles. Although the FOM value (related to resolution/detection limit) is a key parameter for the RI sensors, some other characteristics (detection range, sample volume, cost-effective ratio, portability and capability of integration with other analyzing instruments) are of importance and should be taken into consideration in the specific biochemical applications.

### 3.2. New Areas for Exploration: Volume Sensing

Currently, most RI sensors are developed based on near-field optics, which uses evanescent waves in the subwavelength region. Dramatically decayed evanescence has marked light-analyte interaction, indicating high sensitivity, but its spatial interaction is intrinsically limited by the attenuation characteristics of evanescent waves. On the other hand, some bio-analytes (e.g., eukaryotic cells) tend to measure 20–30 μm in diameter; in this case, organisms deep inside the cells are outside of the evanescent field and cannot be measured accurately. For RI sensors working on evanescent waves, only the sample in contact with the sensing surface can be measured, which hinders the detection of naturally suspended samples. Furthermore, because the spatial measurement range is expanded from nearly 2D to 3D, volume sensing is a feasible way to increase light-analyte interaction. In volume sensing, the lightwave can completely permeate the targeted sample and detect every particle in the solution—not only the sample attached to the sensing surface. Hence, this method is particularly useful for monolithic biological samples (eukaryotic cells and tissues) and analytes in low-concentration solutions, which is a rising research subject offering clear advantages in a host of applications. 

Fabry–Pérot (FP) etalon is considered to be a promising choice for the volume sensing application due to its simple structure and growing performance [16]. Some recent research demonstrated that open-access optical cavities with high Q factor and low mode volume can be achieved by utilizing micro-scale curved-mirrors [55], or even a spherical mirror on a fiber tip-end and an assorted planar mirror [56]. Since RI of living kidney cells [22,57,58], these high performance FP RI sensors are inspired to have bright future in studying cell physiology and pathology.

### 3.3. Advanced Methods for Performance Enhancement

According to Equation (1), FOM can be optimized from two angles: sensitivity and quality factor. Improving sensitivity depends mainly on enhancing the light-analyte interaction; however, strong light-analyte interaction also indicates intense absorption (i.e., a low Q factor) from solvent, where are often water or phosphate-buffered solution with certain concentration. Therefore, spatially accumulating or attracting interested particles to the area with the strongest light-analyte interaction via external field/force (dielectrophoretic [47], ultrasonic, and magnetic methods [59]) or a microfluidic sorting structure is an effective way to avoid the influence of solvent absorption. Furthermore, cascaded/hybrid structures, such as coupled resonator-induced transparence (CRIT) [60,61,62] and Vernier effect [26], improve the Q factor and sensitivity synchronously and contribute to resolving the interaction-absorption dilemma.

## 4. Conclusions

This review article has summarized the prominent designs for RI sensing using optofluidic technology. Excellent performance of intelligent designs mentioned in paper indicates a prosperous prospect of optofluidics in bio/chemical analysis. The synergy of photonics and microfluidics offers a great opportunity to achieve the device’s performance improvements and functional extension. And microfluidics technology facilitates devices with portable and cost-effective features, providing steady motivation for their commercialization promotion. In future, more and more subtle and powerful devices will be developed to meet the growing need of measurement in biomedical applications. 

## Figures and Tables

**Table 1:** Critical parameters of the refractive index (RI) sensors based on different principles.

Working Principle	Sensitivity	Q Factor	FOM	Detection Limit	Analyte	Reference
PCF	8699 nm/RIU	-	-	4.0 × 10^−6^ RIU	-	[30]
PCF	1145 nm/RIU	-	-	-	-	[31]
POW	260 nm/RIU	800	-	-	-	[37]
POW	1920 nm/RIU	-	-	5.2 × 10^−7^ RIU	-	[38]
WGM	1.84 pm/mM	4 × 10^5^	-	0.035 mM	Glucose	[42]
WGM	0.018 pm/mg m^−3^	11,500	-	6.9 ppm	Benzene	[43]
SPR	-	-	-	1 pM	BSA	[47]
SPR	~104 nm/RIU	-	-	-	-	[49]
FPC	960 nm/RIU	600	18.79	0.01 RIU	-	[53]
FPC	907 nm/RIU	400	9	1.7 × 10^−5^ RIU	-	[54]
